# Cumulative incidence trends of selected cancer sites in a Philippine population from 1983 to 2002: a joinpoint analysis

**DOI:** 10.1038/sj.bjc.6605640

**Published:** 2010-04-06

**Authors:** V M Medina, A Laudico, M R Mirasol-Lumague, H Brenner, M T Redaniel

**Affiliations:** 1 College of Public Health, University of the Philippines-Manila, 625 Pedro Gil Street, Ermita 1000 Manila, Philippines; 2 Philippine Cancer Society-Manila Cancer Registry, 310 San Rafael St, San Miguel, 1005 Manila, Philippines; 3 Department of Surgery, Philippine General Hospital, University of the Philippines-Manila, Taft Avenue, 1000 Manila, Philippines; 4 Department of Health-Rizal Cancer Registry Rizal Medical Center, Pasig Boulevard, 1600 Pasig City, Philippines; 5 Division of Clinical Epidemiology and Aging Research, German Cancer Research Center, Bergheimer Street 20, D-69115, Heidelberg, Germany

**Keywords:** cancer incidence trends, Philippines, joinpoint analysis

## Abstract

**Background:**

Few studies have investigated incidence trends in the Philippines.

**Methods:**

From the databases of the Manila Cancer Registry, cumulative cancer incidence rates were determined for the five most common cancers for both sexes combined. Using joinpoint analysis, incidence trends for 1983–2002 were estimated.

**Results:**

Among females, increasing trends were found for breast, 5% annual change, lung (0.5%) and colorectal (1.5%) cancers. Decreasing trends were found for cancers of the liver (−1.2%) and cervix (−1.9%). Among males, increasing trends were found for lung cancer (0.5%), whereas liver cancer rates have been decreasing (−1.0%). Colorectal cancer rates fluctuated.

**Conclusion:**

Certain sites showed declining incidence trends, but incidence trends for lifestyle-related cancers continue to rise. The prevention of infection-related cancers should also receive priority, particularly by vaccination programmes.

In recent decades, cancer has slowly become one of the leading causes of morbidity and mortality in the Philippines (DOH, 1962, 2002). Although cancer incidence has been monitored and reported, with updates carried out every 5 years (Parkin *et al*, 1992, 1997, 2002; Curado *et al*, 2007), few studies have investigated trends. In developing countries such as the Philippines, the dearth of population-based incidence data renders the analysis of trends difficult.

Fluctuating incidence rates have been noted in the age-standardised rates (ASRs) of cancer for both males and females since 1992 (Parkin *et al*, 1992, 1997, 2002; Curado *et al*, 2007). Incidence largely reflects the population's exposure to relevant risk factors and the effect of implemented interventions. Any appreciable change in exposure to risk factors would change the incidence rates. Similarly, the presence of effective cancer prevention programmes may allow the prospect of reduction in incidence.

Our study aims to determine the incidence trends of selected cancer sites in an urban Philippine population from 1983 to 2002, using joinpoint regression analysis.

## MATERIALS AND METHODS

Data for the selected cancer sites were obtained from the Philippine Cancer Society-Manila Cancer Registry (PCS-MCR) for the period 1983–2002. The PCS-MCR actively identifies cases from the 169 hospitals in the National Capital Region (NCR) and its surrounding areas, and from four local civil registry offices in the four major cities of the NCR (Manila, Quezon, Pasay and Caloocan). It covers approximately 7% of the population of the Philippines, or some 6 million out of the total population of 88.57 million (National Statistics Office, 2007). The five most common cancer sites based on the number of cases for both sexes combined for the period 1983–2002 were included in the study, namely, colorectal (C18-21), liver (C22), lung (C33-34), breast (C50) and cervix (C53) cancers.

The PCS-MCR follows data collection procedures and uses the cancer registration definitions and guidelines set by the International Association for Research on Cancer (IARC) and the International Association of Cancer Registries (IACR) (International Association of Cancer Registries and International Agency for Research on Cancer, 2004). Registry data were coded using the International Classification of Disease (ICD) in Oncology (ICD-O, 3rd Ed) (Fritz *et al*, 2000) and the ICD-10 (ICD-10) (WHO, 2007), and were encoded using CanReg software (http://www.iacr.com.fr/suppreg.htm) (Cooke *et al*, 2006). It is regarded as among the high-quality cancer registries from developing countries and has been regularly included in Cancer Incidence in Five Continents (Parkin *et al*, 1992, 1997, 2002; Curado *et al*, 2007).

Incidence data included only the number of incident cases among residents of the cities of Manila, Pasay, Caloocan and Quezon, stratified by 5-year age groups. Identifying variables were not included in the study database.

### Data analysis

Cumulative incidence rates were calculated for cancer patients aged 0–74 years, by sex and cancer site. Cumulative rates are a special form of standardised rates in which equal weights are given for all 5-year age groups up to a defined upper age limit, which in this case is 75 years (Esteve *et al*, 1994). For each year included in the study, age-specific rates were computed and multiplied by the length of the age group in years (5 years). These were then summed to derive the cumulative incidence rates.

To determine changes in temporal trends, if any, joinpoint analysis was used (Kim *et al*, 2000). Possible changes in trends were assessed by identifying best-fitting points in which a significant change could have occurred. To describe the linear trends, the estimated annual percentage change (APC) was computed for each of the time periods determined in the analysis, as demarcated by the joinpoints, using the log-linear model. The Joinpoint programme from the US Surveillance, Epidemiology and End Results (SEER) Program was used in the analysis (National Cancer Institute, 2008).

## RESULTS

**Figure 1:**
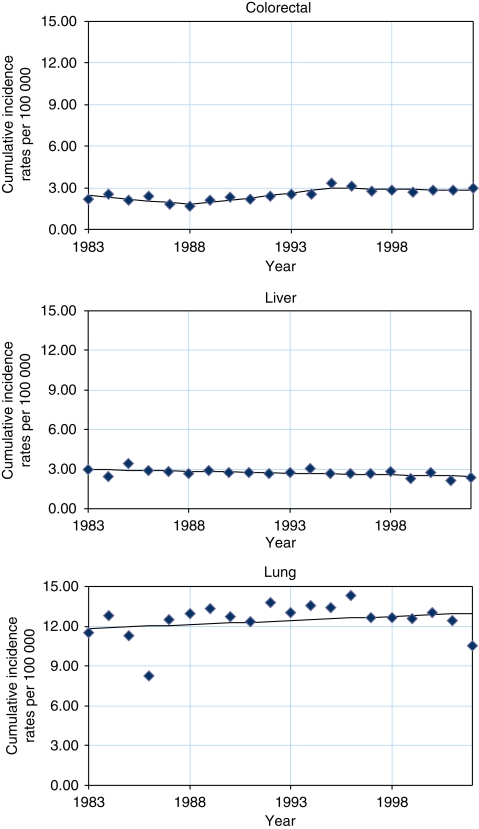
Joinpoint analysis of selected cancer sites in males, 0–74 years old, in a Philippine population (1983–2002).

**Figure 2:**
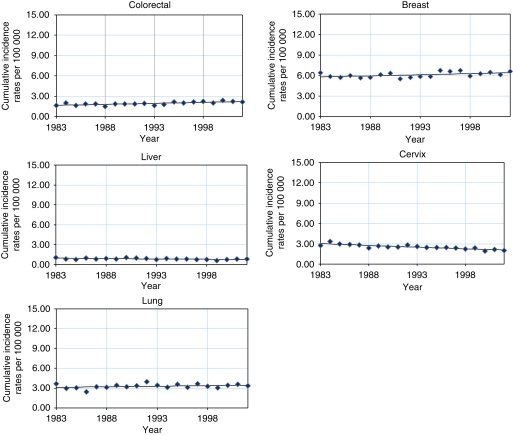
Joinpoint analysis of selected cancer sites in females, 0–74 years old, in a Philippine population (1983–2002).

Tables 1 and 2 and Figures 1 and 2 show the truncated cumulative rates at 0–74 years and the results of the joinpoint analysis. Among males, increasing trends were found for cancer of the lung (APC 0.5%) from 1983 to 2002. Statistically significant decreasing trends were observed for cancer of the liver (APC −1.0%). Fluctuating rates were found for cancer of the colorectum (APC −5.5, 7.1 and −1.0%), in which trends for 1983–1988 and 1988–1995 showed a statistically significant decrease and increase, respectively. Among females, increasing trends were found for cancers of the colorectum (APC 1.5%), lung (APC 0.5%) and breast (APC 0.5%). Statistically significant decreasing trends were found in cancers of the liver (APC −1.2%) and cervix (APC −1.9%).

## DISCUSSION

To our knowledge, this is the first comprehensive analysis of cancer incidence trends in the Philippines over the past few decades using joinpoint regression. Rather divergent trends were observed for various cancer sites in both sexes, which might reflect major changes in risk factors and preventive factors over time. The increase in the incidence of cancers of the breast and colorectum may indicate increasing trends in unhealthy lifestyle factors, including increasing alcohol consumption, sedentary lifestyle and unhealthy diet. This probably reflects the adoption of a Western lifestyle that accompanied the rapid urbanisation of the Greater Manila Area and of the surrounding areas (Harpham and Stephens, 1991; JBIC *et al*, 2004). Trends in breast cancer incidence may also have been affected by changes in fertility patterns (Laudico *et al*, 2009).

From 1970 to 1972 and from 1994 to 1996, per capita alcohol consumption in the Philippines increased by >50% (WHO, 1999). In 2001, the reported rate of regular drinking, defined as drinking ⩾4 days per week, was 11.1, 13 and 5.9% for both sexes combined, males and females, respectively (Tiglao *et al*, 2001). Lung cancer showed an increasing trend in both sexes, reflecting the increasing per capita consumption of cigarettes from 1990 in 1970 to 2160 in 1980 (Shafey *et al*, 2003); this later decreased to 1846 in 1990 and to 1462 in 1995 (Shafey *et al*, 2003), but the full effects on cancer incidence are likely to be observed only in the next few decades.

The prevalence of obesity is reported to have increased in the decade 1993–2003 (Food and Nutrition Research Institute, 1993; Dans *et al*, 2005), and protein and energy intake levels have continued to increase (UN FAO, 2006), whereas a decreasing trend in the consumption of fruits and vegetables was reported (Food and Nutrition Research Institute, 1993; Dans *et al*, 2005). A majority of males (57.4%) and females (57.0%) were reported as being physically inactive (Baltazar *et al*, 2004). Among young people, continued modernisation has resulted in a decrease in such physical activities as household chores and active commuting (Tudor-Locke *et al*, 2003). Advancements in transportation, the introduction of portable gaming devices and reduction in laborious work suggest that inactivity in the Philippines will increase further.

The improvements in infectious disease control and the introduction of vaccines in recent years may have reduced the incidence of infection-related cancers, such as cervix cancer. The promotion of family planning in the 1970s (Williamson *et al*, 1983) may have led to a reduction in unsafe sexual practices and limited HPV transmission. In 1971, Republic Act No. 6365 was enacted, which provided for the establishment of clinics, providing family planning education and services as part of overall health care and making available all methods of contraception, except abortion. Although condom use was reported to be low (National Statistics Office, 1998), Filipino sexual practices are still conservative, largely because of the influence of the Catholic Church, which permits only sex within a monogamous marriage, making high-risk practices such as polygamy, pre- and extramarital sex socially unacceptable (Francoeur and Noona, 2001). However, more investigation is needed to determine other possible causes for the decreasing trend.

In the Philippines, the first HBV vaccines were made available in 1982. The decrease in liver cancer incidence may be partly attributable to the ‘mass vaccination’ among those who could afford it, which started in the mid 1980s. At present, HBV vaccine is widely available in the country but no community-wide vaccination programme has yet been implemented. Aside from alcohol consumption, which is increasing, little information is available on the prevalence of other risk factors for liver cancer, and more investigation is needed.

In the interpretation of our study, several limitations are relevant. Our results reflect cancer incidence only in the four major cities of the NCR, and they are not necessarily representative of the whole country. In particular, westernisation of lifestyle, health education and access to preventive measures are more prevalent in these cities than in other areas.

In conclusion, declining cancer incidence trends have already been observed in some sites, particularly those that are infection related (liver and cervix). However, incidence trends for lifestyle-related cancers continue to rise and highlight the importance of interventions targeting lifestyle-related risk factors. Although present intervention programmes aim to reduce unhealthy dietary habits, alcohol drinking and physical inactivity, their effectiveness can only be evaluated in the future. The prevention of infection-related diseases such as liver and cervical cancers receives priority, particularly by the vaccination programmes.

## Figures and Tables

**Table 1:** Joinpoint analysis of selected cancer sites in males, 0–74 years old, in a Philippine population (1983–2002)

		**Trend 1**								**Trend 2**								**Trend 3**							
		**Years**		**Cumulative rates**						**Years**		**Cumulative rates**						**Years**		**Cumulative rates**					
**Cancer site**	***n***	**Period begin**	**Period end**	**Period begin**	**Period end**	**APC**	**95% CI**			**Period begin**	**Period end**	**Period begin**	**Period end**	**APC**	**95% CI**			**Period begin**	**Period end**	**Period begin**	**Period end**	**APC**	**95% CI**		
Colorectal	4528	1983	1988	2.2	1.7	−5.5	−10.1	—	−0.6	1988	1995	1.7	3.3	7.1	3.1	—	11.3	1995	2002	3.3	2.9	−1.0	−3.9	—	2.1
Liver	4177	1983	2002	3.0	2.4	−1.0	−1.7	—	−0.3	—	—	—	—	—	—	—	—	—	—	—	—	—	—	—	—
Lung	8450	1983	2002	11.5	10.6	0.5	−0.5	—	1.5	—	—	—	—	—	—	—	—	—	—	—	—	—	—	—	—

Abbreviations: APC=annual percent change; 95% CI=95% confidence interval.

**Table 2:** Joinpoint analysis of selected cancer sites in females, 0–74 years old, in a Philippine population (1983–2002)

		**Trend 1**							
		**Years**		**Cumulative rates**					
**Cancer site**	***n***	**Period begin**	**Period end**	**Period begin**	**Period end**	**APC**	**95% CI**		
Colorectal	4251	1983	2002	1.7	2.1	1.5	0.8	—	2.3
Liver	1538	1983	2002	1.1	0.8	−1.2	−2.1	—	−0.3
Lung	2807	1983	2002	3.6	3.3	0.5	−0.3	—	1.4
Breast	12 908	1983	2002	6.4	6.6	0.5	0.1	—	1.0
Cervix	5583	1983	2002	2.7	2.0	−1.9	−2.5	—	−1.4

Abbreviations: APC=annual percent change; 95% CI=95% confidence interval.

## References

[bib1] Baltazar JC, Ancheta CA, Aban IB, Fernando RE, Baquilod MM (2004) Prevalence and correlates of diabetes mellitus and impaired glucose tolerance among adults in Luzon, Philippines. Diabetes Res Clin Pract 64: 107–115 15063603 10.1016/j.diabres.2003.10.013

[bib2] Cooke A, Parkin D, Ferlay J (2006) Canreg4 version 4.33. International Agency for Research on Cancer: Lyon http://www.iacr.com.fr/suppreg.htm

[bib3] Curado M, Edwards B, Shin H, Storm H, Ferlay J, Heanue M, Boyle P (eds) (2007) Cancer Incidence in Five Continents IARC Scientific Publications No. 160. IARC: Lyon

[bib4] Dans A, Morales D, Velandria F, Abola T, Roxas Jr A, Punzalan FE, Sy RA, Paz-Pacheco E, Amarillo L, Villaruz MV (2005) National Nutrition and Health Survey (NNHeS): atherosclerosis-related diseases and risk factors. Philipp J Inter Med 43: 103–115

[bib5] DOH (1962) Philippine Health Statistics. Department of Health: Manila

[bib6] DOH (2002) Philippine Health Statistics. Department of Health: Manila

[bib7] Esteve J, Benhamou E, Raymond L (1994) Statistical Methods in Cancer Research. Volume IV - Descriptive Epidemiology Vol. IV. International Agency for Research on Cancer: Lyon 7698823

[bib8] Food and Nutrition Research Institute (1993) National Nutrition Survey. Food and Nutrition Research Institute: Manila

[bib9] Francoer R, Noonan R. (2001). The International Encyclopedia of Sexuality. Continuum: New York.

[bib10] Fritz A, Percy C, Jack A, Shanmugaratnam K, Lobin L, Parkin DM, Whelan S (2000) International Classification of Diseases for Oncology 3rd edn

[bib11] Harpham T, Stephens C (1991) Urbanization and health in developing countries. World Health Stat Q 44: 62–69 1926894

[bib12] International Association of Cancer Registries, International Agency for Research on Cancer (2004) Guidelines on Confidentiality for Population-Based Cancer Registries, Internal Report No. 2004/03. IARC: Lyon

[bib13] JBIC, ADB, WB (2004) Infrastructure in East Asia: The Way Forward. Japan Bank for International Cooperation, Asian Development Bank, World Bank Joint Study

[bib14] Kim HJ, Fay MP, Feuer EJ, Midthune DN (2000) Permutation tests for joinpoint regression with applications to cancer rates. Stat Med 19: 335–351 10649300 10.1002/(sici)1097-0258(20000215)19:3<335::aid-sim336>3.0.co;2-z

[bib15] Laudico A, Redaniel MT, Mirasol-Lumague MR, Mapua C, Uy G, Pukkala E, Pisani P (2009) Epidemiology and clinicopathology of breast cancer in Metro Manila and Rizal Province, Philippines. Asian Pacific J Cancer Prev 10: 165–170 19469648

[bib16] National Cancer Institute (2008) Joinpoint Regression Program Version 3.3. National Cancer Institute Statistical Research and Application: United States, 〈http://srab.cancer.gov/joinpoint/〉

[bib17] National Statistics Office (1998) National Demographic and Health Survey. National Statistics Office: Manila

[bib18] National Statistics Office (2007) Census of Population, Report No. 1-N (NCR)- Population by Province, City/Municipality and Barangay. National Statistics Office: Manila

[bib19] Parkin D, Muir C, Whelan S, Gao Y, Ferlay J, Powell J (1992) Cancer Incidence in Five Continents Vol. VI. International Agency for Research on Cancer: Lyon

[bib20] Parkin D, Whelan S, Ferlay J, Raymond L, Young J (1997) Cancer Incidence in Five Continents Vol. VII. International Agency for Research on Cancer: Lyon

[bib21] Parkin D, Whelan S, Ferlay J, Teppo L, Thomas D (2002) Cancer Incidence in Five Continents Vol. VIII. International Agency for Research on Cancer: Lyon

[bib22] Shafey O, Dolwick S, Guindon GE (eds) (2003) Tobacco Control Country Profiles 2nd edn American Cancer Society: Atlanta

[bib23] Tiglao TV, Baltazar JC, Baquilod MM (2001) Baseline Behavioral Risk Factor Survey Philippines. Department of Health: Manila

[bib24] Tudor-Locke C, Ainsworth BE, Adair LS, Popkin BM (2003) Physical activity in Filipino youth: the Cebu Longitudinal Health and Nutrition Survey. Int J Obes Relat Metab Disord 27: 181–190 12586997 10.1038/sj.ijo.802207

[bib25] UN FAO (2006) Compendium of Food and Agriculture Indicators – 2006. UN Food and Agriculture Organization (FAO): Rome http://www.fao.org/statistics/compendium_2006/list.asp

[bib26] WHO (1999). World Health Organization: Geneva

[bib27] WHO (2007) International Classification of Diseases 10 online. World Health Organization: Geneva, http://www.who.int/classifications/icd/en/

[bib28] Williamson NE, Parado J, Maturan E (1983) Providing maternal and child-health family planning services to a large rural population: results of the Bohol project. Am J Public Health 73: 62–71 6848001 10.2105/ajph.73.1.62 PMC1650447

